# Serotypes, Antibiotic Susceptibilities, and Multi-Locus Sequence Type Profiles of *Streptococcus agalactiae* Isolates Circulating in Beijing, China

**DOI:** 10.1371/journal.pone.0120035

**Published:** 2015-03-17

**Authors:** Ping Wang, Jing-jing Tong, Xiu-hua Ma, Feng-li Song, Ling Fan, Cui-mei Guo, Wei Shi, Sang-jie Yu, Kai-hu Yao, Yong-hong Yang

**Affiliations:** 1 Key Laboratory of Major Diseases in Children and National Key Discipline of Pediatrics, Ministry of Education, Beijing Pediatric Research Institute, Beijing Children’s Hospital, Capital Medical University, Beijing, 100045, China; 2 Department of Obstetrics, People’s Hospital of Beijing Daxing District, Capital Medical University, Beijing, 102600, China; 3 Department of Obstetrics, Beijing Obstetrics and Genecology Hospital, Capital Medical University, Beijing, 100026, China; Rockefeller University, UNITED STATES

## Abstract

**Background:**

To investigate the serotypes, antibiotic susceptibilities, and multi-locus sequence type (MLST) profiles of *Streptococcus agalactiae* (*S*. *agalactiae*) in Beijing to provide references for the prevention and treatment of *S*. *agalactiae* infections.

**Methods:**

All isolates were identified using the CAMP test and the latex-agglutination assay and serotyped using a Strep-B-Latex kit, after which they were assessed for antibiotic susceptibility, macrolide-resistance genes, and MLST profiles.

**Results:**

In total, 56 *S*. *agalactiae* isolates were identified in 863 pregnant women (6.5%). Serotypes Ia, Ib, II, III, and V were identified, among which types III (32.1%), Ia (17.9%), Ib (16.1%), and V (14.3%) were the predominant serotypes. All isolates were susceptible to penicillin and ceftriaxone. The nonsusceptiblity rates measured for erythromycin, clarithromycin, azithromycin, telithromycin, clindamycin, tetracycline, and levofloxacin were 85.7%, 92.9%, 98.2%, 30.4%, 73.2%, 91%, and 39.3%, respectively. We identified 14 sequence types (STs) for the 56 isolates, among which ST19 (30.4%) was predominant. The rate of fluoroquinolone resistance was higher in serotype III than in the other serotypes. Among the 44 erythromycin-resistant isolates, 32 (72.7%) carried *ermB*.

**Conclusion:**

*S*. *agalactiae* isolates of the serotypes Ia, Ib, III, and V are common in Beijing. Among the *S*. *agalactiae* isolates, the macrolide and clindamycin resistance rates are extremely high. Most of the erythromycin-resistant isolates carry *ermB*.

## Introduction

*Streptococcus agalactiae* (Group B *streptococcus*, *GBS*) is an opportunistic pathogen that colonizes the lower digestive and urogenital tracts of healthy people and can be isolated from the genitourinary and gastrointestinal tracts of up to 35% of healthy adults [1–2]. In the last 40 years, *S*. *agalactiae* has been the most common pathogen responsible for maternal and neonatal infections. An estimated 20–30% of women are colonized by GBS in developed countries; however, there is a paucity of data regarding GBS colonization in the developing world [3–5]. Maternal intrapartum *S*. *agalactiae* colonization is a primary risk factor for early-onset *S*. *agalactiae* infection in infants. In the absence of any intervention, 1–2% of infants born to *S*. *agalactiae*-colonized mothers are estimated to develop early-onset *S*. *agalactiae* infection, including neonatal pneumonia, sepsis, and meningitis [6–8]. Severe *S*. *agalactiae* infections can result in neonatal mortality or permanent disability [9].

In 1996, the US Centers for Disease Control and Prevention (CDC) published guidelines for the prevention of perinatal *S*. *agalactiae* infection [10]. The first strategy, intrapartum antibiotic prophylaxis, is offered to women identified as *S*. *agalactiae* carriers using prenatal screening cultures prepared at 35–37 weeks of gestation and to women who develop premature onset of labor or rupture of membranes at <37 weeks of gestation. The second strategy, intrapartum antibiotic prophylaxis, is provided to women who, at the time of labor or membrane rupture, develop one or more risk conditions (e.g., <37 weeks of gestation, a membrane-rupture duration of ≥18 h, a temperature of ≥38.0°C, *S*. *agalactiae* bacteriuria during pregnancy, or previous delivery of an infant who later developed *S*. *agalactiae* infection). These guidelines were updated and republished in 2002 and 2010 [11–12]. The new guidelines recommend prenatal screening for vaginal and rectal *S*. *agalactiae* colonization in all pregnant women at 35–37 weeks of gestation, and intrapartum antibiotic prophylaxis is offered to women identified as *S*. *agalactiae* carriers or to women who develop one or more risk conditions. Moreover, an updated algorithm has been developed for the management of newborns exposed to intrapartum antibiotic prophylaxis. Universal screening and the use of intrapartum antibiotic prophylaxis has led to substantial reductions in the number of early-onset *S*. *agalactiae* infections in newborns [10–12].

Capsular polysaccharide (CPS) is a major virulence factor of *S*. *agalactiae* that enables the bacterium to evade the host innate defense mechanisms [13]. Currently, 10 CPS types are recognized: serotypes Ia, Ib, and II–IX [14]. An epidemiological study showed that *S*. *agalactiae* serotypes Ia, II, III, and V are the predominant serotypes in invasive neonatal infections, accounting for >90% of all *S*. *agalactiae* isolates [1]. In any given country, understanding the serotype distribution of a pathogen is a key prerequisite to formulating serotype-based vaccines. A CPS-based conjugate vaccine that includes serotypes Ia, Ib, II, III, and V, which is currently in development, could potentially prevent neonatal, pediatric, adult, and pregnancy-associated *S*. *agalactiae* infections. It is expected that an effective vaccine will prevent the majority of infant *S*. *agalactiae* infections (both early and late onset), avoid the limitations of intrapartum antibiotic prophylaxis, and be cost effective [15–16].

In this study, we investigated the serotypes, antimicrobial resistance, and molecular epidemiology of 56 *S*. *agalactiae* isolates collected from pregnant women at 2 hospitals in Beijing in 2012–2013. Our aim was to provide a basis for the development of methods to prevent *S*. *agalactiae* infections.

## Materials and Methods

### *S*. *agalactiae* isolates

This study protocol was in accordance with the ethical standards of the responsible regional committee on human experimentation and the Helsinki Declaration of 1975 (revised in 1983). It was approved by the Ethics Committees of the two hospital, Ethic Committee of the Beijing Obstetrics and Genecology Hospital and Ethic Committee of the People’s Hospital of Beijing Daxing District. Only verbal consent for the participants. We did not obtain the written consent, because the verbal consent had been written to the case records for all participants. The ethics committees also approved this consent procedure.

Lower vaginal and rectal swabs were obtained from 469 pregnant women who attended the Beijing Obstetrics and Genecology Hospital and 394 pregnant women who attended the People’s Hospital of Beijing Daxing District from 2012 to 2013. The women were 35–37 weeks pregnant and aged 25–40 years.

### Detection and confirmation of *S*. *agalactiae*

Rectal and vaginal swabs were collected at the same time from each pregnant woman using sterile swabs. All swabs were stored at −80°C and subsequently transferred to the Microbial and Immunology Laboratory of the Beijing Pediatric Research Institute. Because of limitations associated with the particular hospitals, the time interval for the transportation of the samples differed between the two hospitals (one or two weeks for the Beijing Obstetrics and Genecology Hospital and two months for People’s Hospital of Beijing Daxing District). The swabs were inoculated onto tryptone soy agar plates supplemented with 5% sheep blood. The plates were incubated at 35°C under 5% CO_2_ and examined after 18–24 h of culture. To confirm suspected *S*. *agalactiae* colonies based on their morphology, we performed Gram staining, catalase reaction assays, CAMP tests, and Lancefield grouping based on latex agglutination (Streptococcal Grouping Kit, Oxoid, Basingstoke, UK). In total, 56 strains were confirmed to be *S*. *agalactiae*.

### Serotyping by latex agglutination

Serotyping based on latex agglutination and the serotype determination method was performed in accordance with the instructions provided with the Strep-B-Latex Kit (reagents Ia, Ib, and II–IX; Statens Serum Institut, Copenhagen, Denmark) and procedures described recently [17]. The isolate was defined as non-typeable (NT) if the method failed to identify it into any serotype.

### Antimicrobial susceptibility testing

The E-test (AB Biomerileux, Sweden) method was used to determine the minimal inhibitory concentrations (MICs) of the following 7 antibiotics: penicillin, ceftriaxone, erythromycin, azithromycin, clarithromycin, levofloxacin, and clindamycin. The susceptibility of the isolates to tetracycline and telithromycin was tested using the disk-diffusion method. The isolates were grown at 35°C on trypticase soy agar plates supplemented with 5% sheep blood. The grown bacteria were suspended in normal saline, and the suspensions were adjusted to a 0.5 McFarland standard. Sterile cotton swabs were dipped into the bacterial suspensions and used to spread them uniformly on Mueller-Hinton agar (Oxoid) supplemented with 5% sheep blood. The plates were then incubated under 5% CO_2_ for 20–24 h. The criteria of the Clinical and Laboratory Standards Institute (CLSI) 2012 edition were applied for classifying the isolates as susceptible, intermediate, or resistant. We used *S*. *pneumoniae* ATCC 49619 as a quality-control strain in each set of tests to ensure the accuracy of the results. Susceptibility to telithromycin was determined according to the criterion defined for *S*. *pneumoniae* because no criteria are available for assessing the telithromycin susceptibility of *S*. *agalactiae*.

### DNA extraction

Chromosomal DNA was isolated from overnight cultures of isolates grown on 5% tryptone soy agar using a DNA Mini Kit (SBS Genetech, Beijing) according to the manufacturer’s instructions.

### Detection of macrolide-resistance genes

The macrolide resistance genes *ermB* and *mefA* were detected by polymerase chain reaction (PCR), which was performed on erythromycin-nonsusceptible strains. The procedures used in the present study have been described previously [18].

### Multi-locus sequence typing

Seven housekeeping genes (*adhP*, *pheS*, *atr*, *glnA*, *sdhA*, *glcK*, and *tkt*) were PCR-amplified using oligonucleotide primers. The primers and the PCR procedure were implemented as described by Jones et al[19]. The amplification products were sequenced by the Beijing Qing Department of New Biological Technology Co. Ltd. The identified alleles were submitted to the *S*. *agalactiae* MLST database (http://pubmlst.org/sagalactiae/) for designation. Alleles that were not present in the *S*. *agalactiae* MLST database were verified by resequencing of both strands of the gene fragments and then submitted to the database for a new designation. We used eBURST (version 3; http://eburst.mlst.net) to estimate the relationships between the various isolates and to compare these isolates with those present in the MLST database. Various sequence types (STs) were clustered in a clonal complex (CC) only if they shared 6 identical alleles of the 7 MLST loci with another ST in the group.

### Statistical analysis

The data were analyzed using WHONET 5.6 software (http://who.int/drugresistance/whonetsoftware/en/), which is recommended by the World Health Organization. The χ^2^ test was performed using SPSS 17 for statistical comparisons. Differences were considered statistically significant at *P* < 0.05.

## Results

### Carriage rate

The culture preparations revealed that of the swabs obtained from the 863 pregnant women, 56 were positive for *S*. *agalactiae*, with an overall *S*. *agalactiae* carriage rate of 6.5% (56/863). The carriage rate at the Beijing Obstetric and Genecology Hospital was 8.7% (41/469), whereas it was 3.6% (14/394) at the People’s Hospital of Beijing, Daxing District, and this difference was statistically significant (χ^2^ = 9.661, *P* < 0.01).

### Serotype distribution

We identified 5 serotypes in the 56 *S*. *agalactiae* isolates. The distribution of the serotypes was as follows: III, 32.1% (18/56); Ia, 17.9% (10/56); Ib, 16.1% (9/56); V, 14.3% (8/56); and II, 5.4% (3/56). The other 14.3% (8/56) of the isolates were identified as NT.

### Antimicrobial susceptibility

The susceptibility to 9 antibiotics, the antibiotic MIC in the 56 *S*. *agalactiae* isolates and a comparison of the serotypes are listed in Table 1. Based on the CLSI 2012 criteria, all isolates were susceptible to both penicillin and ceftriaxone. The highest penicillin MIC measured in this study was 0.125 μg/mL.

**Table 1:** Susceptibility to 9 antibiotics, antibiotic minimal inhibitory concentrations (MICs), and serotypes of 56 *Streptococcus agalactiae* isolates.

Antibiotic^a^	Non-susceptibility and MIC^b^	All isolates	Serotypes					
			Ia (n = 10)	Ib (n = 9)	II (n = 3)	III(n = 18)	V (n = 8)	NT (n = 8)
PEN	MIC50 (μg/mL)	0.094	0.094	0.094	0.064	0.094	0.094	0.094
	MIC90 (μg/mL)	0.125	0.125	0.125	0.094	0.125	0.125	0.125
	MIC range (μg/mL)	0.008–0.125	0.064–0.125	0.064–0.125	0.047–0.094	0.032–0.125	0.008–0.125	0.064–0.125
CRO	MIC50 (μg/mL)	0.094	0.125	0.125	0.125	0.064	0.125	0.094
	MIC90 (μg/mL)	0.19	0.19	0.19	0.19	0.125	0.5	0.5
	MIC range (μg/mL)	0.047–0.5	0.047–0.25	0.064–0.19	0.064–0.19	0.064–0.19	0.094–0.5	0.064–0.5
ERY	I (%)	7.1	10.0	0.0	0.0	5.6	0.0	12.5
	R (%)	78.6	90.0	100.0	100.0	72.2	75.0	62.5
	MIC50 (μg/mL)	16	12	≥256	≥256	32	16	4
	MIC90 (μg/mL)	≥256	≥256	≥256	≥256	≥256	≥256	≥256
	MIC range (μg/mL)	0.125–≥256	0.38–≥256	≥256	≥256	0.125–≥256	0.125–≥256	0.19–≥256
CLR	I (%)	14.3	20.0	0.0	100.0	16.7	12.5	25.0
	R (%)	78.6	80.0	100.0	.0	72.2	75.0	62.5
	MIC50 (μg/mL)	≥256	12.0	≥256	≥256	≥256	64	8
	MIC90 (μg/mL)	≥256	≥256	≥256	≥256	≥256	≥256	≥256
	MIC range (μg/mL)	0.125–≥256	0.5–≥256	≥256	≥256	0.125–≥256	0.25–≥256	0.25–≥256
AZM	I (%)	10.7	0.0	0.0	0.0	11.1	0.0	12.5
	R (%)	87.5	100.0	100.0	100.0	88.9	87.5	87.5
	MIC50 (μg/mL)	≥256	48	≥256	≥256	≥256	32	32
	MIC90 (μg/mL)	≥256	≥256	≥256	≥256	≥256	≥256	≥256
	MIC range (μg/mL)	0.5–≥256	2–≥256	64–≥256	≥256	1–≥256	0.5–≥256	1–≥256
CLI	I (%)	8.9	30.0	0.0	0.0	0.0	0.0	12.5
	R (%)	64.3	40.0	100.0	100.0	72.2	50.0	50.0
	MIC50 (μg/mL)	≥256	0.5	≥256	≥256	≥256	0.19	0.38
	MIC90 (μg/mL)	≥256	≥256	≥256	≥256	≥256	≥256	≥256
	MIC range (μg/mL)	0.125–≥256	0.125–≥256	≥256	≥256	0.125–≥256	0.125–≥256	0.19–≥256
LVX	I (%)	3.6	20.0	0.0	0.0	0.0	0.0	0.0
	R (%)	35.7	10.0	44.4	0.0	72.2	12.5	12.5
	MIC50 (μg/mL)	1.5	1.5	2	0.75	≥32	0.75	1
	MIC90 (μg/mL)	≥32	6	≥32	1	≥32	≥32	≥32
	MIC range (μg/mL)	0.25–≥32	0.38–≥32	0.75–≥32	0.75–1	0.75–≥32	0.25–≥32	0.75–≥32
TCY^c^	I (%)	7.1	0.0	33.3	0.0	0.0	0.0	12.5
	R (%)	83.9	100.0	33.3	100	100	87.5	75.0
TLT^c^	I (%)	26.8	20.0	33.3	0.0	33.3	37.5	12.5
	R (%)	3.6	0.0	11.1	33.3	0.0	0.0	0.0

a: PEN, penicillin; CRO, ceftriaxone; ERY, erythromycin; AZM, azithromycin; CLR, clarithromycin; CLI, clindamycin; LVX, levofloxacin; TCY, tetracycline; and TLT, telithromycin.

b: I, intermediate; R, resistant; MIC, minimal inhibitory concentrations.

c: TCY and TLT were tested using the disk-diffusion method.

The *S*. *agalactiae* strains’ resistance rates for erythromycin, azithromycin, clarithromycin, telithromycin, tetracycline, and clindamycin were estimated to be 78.6%, 87.5%, 78.6%, 3.6%, 83.9%, and 64.3%, respectively. Among the isolates, 48.2% (27/56) showed MICs values of ≥256 μg/mL for erythromycin and 57.1% (32/56) for azithromycin. Co-resistance between erythromycin and clindamycin was high. All clindamycin-resistant isolates were resistant to erythromycin, whereas 79.5% (35/44) of the strains were also resistant to clindamycin. The cross-resistance between erythromycin and telithromycin was relatively low. Only 4.5% (2/44) of the erythromycin-resistant isolates were resistant to telithromycin, and 61.4% (27/44) were still susceptible to telithromycin.

It was found that 35.7% (20/56) of the *S*. *agalactiae* isolates were resistant to levofloxacin, and the MICs in these isolates were ≥32 μg/mL. The levofloxacin resistance rate of serotype III (72.2%) was significantly higher than that of the other serotypes (χ^2^ = 12.064, *P* < 0.01).

### Detection of macrolide resistance genes

The macrolide resistance genes were determined for the 44 isolates of erythromycin-resistant *S*. *agalactiae* (ERSA), of which 13 (29.5%) contained both *ermB* and *mefA*, 19 (43.2%) were only positive for *ermB*, 10 (22.7%) were only positive for *mefA*, and the remaining 2 contained neither *ermB* nor *mefA* (Table 2). The MICs in the *ermB-*positive ERSA strains were determined to be high at ≥256 μg/mL (81.3%, 26/32). In comparison, 90% (9/10) of the single *mefA*-positive ERSA strains presented lower MICs of ≤16 μg/mL.

**Table 2:** Distribution of macrolide resistance genes and MICs in erythromycin-resistant *S*. *agalactiae* (ERSA).

Macrolide resistance genes		n (%)	Erythromycin MIC^a^ (μg/mL) distribution (n)				
ermB	mefA		1~8	~16	~32	~128	≥256
+	+	13 (29.5%)	1	1	1	0	10
+	-	19 (43.2%)	2	1	0	0	16
-	+	10 (22.7%)	4	5	0	0	1
-	-	2 (4.5%)	1	1	0	0	0

a: MIC, minimal inhibitory concentrations.

### MLST

MLST analysis revealed the presence of 14 STs among the 56 *S*. *agalactiae* isolates. We identified 2 new STs, ST652 and ST653. Eight ST types were identified in ≥3 isolates, which covered 50 isolates (89.3%). ST19 was the most prevalent type (30.4%), followed by ST23 (10.7%), ST12 (10.7%), ST1 (10.7%), ST485 (7.1%), ST17 (7.1%), ST10 (7.1%), and ST652 (5.4%). Other types, including ST8, ST24, ST27, ST28, ST163, and ST653, were also identified in the present study, but only 1 isolate was detected for each (1.8%).

eBURST analysis revealed the presence of 3 CCs and 3 singletons. CC19 (20 isolates, 35.7%), comprising 4 STs, was the most common group. CC12 accounted for 25.0% of the isolates, and CC23 comprised 16.1%.

The relationships between the STs and serotypes are presented in Table 3. One-to-one correspondences were not found between the common serotypes and STs, although the main corresponding relationship was obvious. Further analysis revealed that the levofloxacin resistance rate for the ST19/serotype III isolates was 92.9% (13/14), whereas all of the ST17/serotype III isolates were susceptible to levofloxacin, and 75.0% (15/20) of the levofloxacin-resistant isolates belonged to CC19.

**Table 3:** Relationship between sequence types and serotypes of 56 *S*. *agalactiae* isolates.

CC/ST^a^	n (%)	Serotype					
		Ia	Ib	II	III	V	NT^c^
CC19	20 (35.7)	0	0	3	14	2	1
ST19	17 (30.4)	0	0	0	14	2	1
ST28	1 (1.8)	0	0	1	0	0	0
ST27	1 (1.8)	0	0	1	0	0	0
ST653^b^	1 (1.8)	0	0	1	0	0	0
CC23	8 (16.1)	6	0	0	0	0	2
ST23	6 (10.7)	4	0	0	0	0	2
ST24	1 (1.8)	1	0	0	0	0	0
ST163	1 (1.8)	1	0	0	0	0	0
CC12	14 (25)	0	9	0	0	0	5
ST12	6 (10.7)	0	5	0	0	0	1
ST10	4 (7.1)	0	4	0	0	0	0
ST8	1 (1.8)	0	0	0	0	0	1
ST652^b^	3 (5.4)	0	0	0	0	0	3
ST1	6 (10.7)	0	0	0	0	6	0
ST17	4 (7.1)	0	0	0	4	0	0
ST485	4 (7.1)	4	0	0	0	0	0
Total	56 (100)	10	9	3	18	8	8

a: CC, clonal complex; ST, sequence type;.

b: STs were detected in our study.

c; NT, non-typeable.

## Discussion

Data collected from distinct geographical areas have revealed considerable variation in the phenotypic and genotypic characteristics of *S*. *agalactiae* isolates [20–22]. However, the distribution of *S*. *agalactiae* serotypes and the antimicrobial resistance of *S*. *agalactiae* isolates in China remain poorly investigated. This study adds new information on *S*. *agalactiae* carriage in Beijing, China.

In this study, the *S*. *agalactiae* carriage rate in pregnant women in Beijing was determined to be 6.5%. The carriage rate measured in this study was lower than those determined in the USA, the UK, and Taiwan (21.8%) [23] but is comparable to those reported in Japan (8.2% or 16%), Manila (7.5%), and Korea (8.0%)[11, 24, 25]. In the present study, We found that the carriage rate in one hospital (8.7%) was significantly higher than the other one (3.6%). Risk factors may interpret the rate difference between the two hospitals in relation to the carriage rate, the serotype, genotype and antibiotic resistance patterns. In our study, there were no significant differences for others than carriage rates between the two hospitals. Probably the number of cases was too small. One previous study in Beijing in the 1990s showed no association between *S*. *agalactiae* colonization and <37 weeks of gestation, a membrane rupture duration of ≥18 h, and an intrapartum temperature of ≥38.0°C in a Chinese population [26]. Perhaps the difference in the duration of transportation of the samples have affected the results.

The serotype distribution of the *S*. *agalactiae* isolates in the present study was similar to that reported in Beijing by Lu et al. in 2011–2013 [27], in which the serotype frequencies were as follows: III, 41.8%; Ia, 21.4%; V, 14.9%; Ib, 11.9%; VI, 1.5%; II, 7.0%; and IV, VIII, and NT, 0.5%. However, our results differ from those reported in the 1990s by Shen et al., who showed that serotypes II (33%), III (23%), and Ia (16%) were the predominant serotypes in pregnant and non-pregnant women in Beijing [28]. This difference in distribution could be attributed not only to differences in study materials and methods but also to differences in the *S*. *agalactiae* isolates spreading worldwide.

The epidemiological distribution of *S*. *agalactiae* serotypes can vary depending on several factors, including the geographical region, profile of the population being studied, and source of the bacterial isolates. Four serotypes, Ia, II, III, and V, are the most frequently isolated serotypes in the USA and certain European countries, whereas serotypes VI–IX have rarely been described [23]. The present results for patients in Beijing are similar to data obtained in France and the UK [25, 29] but are distinct from those obtained in Brazil [30]. Our results are also different from those reported in adjacent Japan [10, 31]. Serotypes VIII (35.6%) and VI (24.7%) are the most common serotypes isolated from healthy pregnant women in Kawasaki, whereas the most frequent serotypes isolated in Saitama are V (19.1%), Ib (18.6%), III (16.2%), VI (14.9%), and Ia (14.6%).

Our study confirmed that all *S*. *agalactiae* isolates were susceptible to penicillin and ceftriaxone. The penicillin MIC range was 0.008–0.125 μg/mL in this study, whereas the MIC was reported to be ≤0.06 μg/mL in the late 1990s [27]. Over the years, *S*. *agalactiae* has remained uniformly sensitive to penicillin in Beijing, although isolates exhibiting reduced penicillin susceptibility have been reported since 2008 [32–34].

Erythromycin or clindamycin is recommended for *S*. *agalactiae* intrapartum prophylaxis in penicillin-allergic women at high risk of anaphylaxis. However, increasing rates of resistance to these antibiotics have been detected in several regions of the world, including Europe [35], Asia [36], North America [37–39], and South America [40–41]. The erythromycin resistance rate in Beijing, China was 16% in 1999, and it was 45% in Guangzhou [42–43]. The rates of erythromycin and clindamycin resistance measured in our study are considerably higher than those measured in China 15 years ago. Furthermore, in this study, a high rate of cross-resistance between erythromycin and clindamycin was observed. The rates presented here are substantially higher than those determined using data collected from Europe, the USA, Canada, and other Asian countries [30], in addition to Brazil [44]. In this study, 72.7% of all erythromycin-resistant *S*. *agalactiae* strains carried the gene *ermB*, and this rate is considerably higher than those reported in the USA (13.0%) and Spain (58.1%) but is similar to those reported in Japan (87.0%) and Taiwan (73.4%) [23].

Previous studies have shown that telithromycin is highly active against erythromycin-resistant strains, regardless of the mechanism of macrolide resistance [45–46]. Similar results were also found in our study. Telithromycin belongs to a new, semisynthetic, 14-membered ring macrolide class of antibiotics, and it is a derivative that exhibits enhanced activity against macrolide-resistant streptococci [45]. Previous studies of *S*. *pneumoniae* confirmed that telithromycin has a high level of antibacterial activity and that it exhibits strong affinity for ribosomes that enables it to overcome common macrolide resistance mechanisms, including target modification directed by *ermB*-encoded methylase, which methylates A2058, and mutations in the 23S rRNA gene and ribosomal proteins that interrupt macrolide binding [47–48]. The susceptibility to telithromycin was determined using criteria developed for *S*. *pneumoniae;* therefore, there may be slight deviations in our data. Additional research is required to demonstrate that *S*. *agalactiae* is more susceptible to telithromycin than to other macrolide antibiotics.

The first fluoroquinolone-resistant *S*. *agalactiae* was reported in 2003 in Japan with a low prevalence [49]. In this study, the levofloxacin nonsusceptibility rate was 39.3%, whereas the resistance rate was 35.7%. These results are similar to those of a previous study in which the levofloxacin-resistance rate of *S*. *agalactiae* isolates was 37.7% [14], which was considerably higher than the rates reported in Taiwan (4.8%) and Japan (18.4%) [23]. Quinolones are not typically used for treating *S*. *agalactiae* infections in pregnant women and newborns in China, but they are widely used for both clinical and agricultural purposes. The continuous and widespread use of quinolones could have elevated the resistance of various pathogens, including *S*. *agalactiae*, to these drugs. In this study, we observed a high prevalence of fluoroquinolone-resistant *S*. *agalactiae* associated with the levofloxacin-resistant ST19/serotype III clone.

At least 2 major lineages of *S*. *agalactiae* isolates of serotype III, ST17 and ST19, have been reported. ST19/serotype III has been reported as the predominant clone in Canada [50] and the USA [51], whereas ST23/serotype Ia and ST17/serotype III are the most prevalent clones in Italy [52], Portugal [53], and Spain [54]. However, in the Mediterranean region, ST19 is associated with serotypes Ib, II, III, and IV, which could be ascribed to the horizontal transfer of capsular genes among distinct clones [14]. A previous study found that ST17 is strongly associated with neonatal invasive infections [55]. However, it is difficult at present to conclude that the low prevalence of neonatal *S*. *agalactiae* infections in Beijing is associated with limited carriage of ST17.

The improved use of intrapartum antimicrobial prophylaxis has resulted in a substantial reduction in early-onset *S*. *agalactiae* infections, but it is unlikely to prevent most late-onset neonatal infections. *S*. *agalactiae* infections continue to be a major cause of illness and death among newborns. Widespread antimicrobial use also increases the risk of the emergence of antimicrobial-resistant organisms. The most promising strategy for preventing neonatal *S*. *agalactiae* infections is to vaccinate women of childbearing age. Our results indicate that inclusion of serotypes Ia, Ib, II, III, and V in an *S*. *agalactiae* vaccine could provide protection against the development of this infection in neonates in Beijing, China.

To date, no routine screening or antibiotic resistance testing of *S*. *agalactiae* has been conducted, and no standard prevention measure has been formulated for pregnant women in China. Although the *S*. *agalactiae* carriage rate and the prevalence of neonatal invasive infection are relatively low in China, neonatal invasive infection could be prevented effectively by following a defined standard procedure. Screening and prophylaxis for this infection should therefore be considered in China. Furthermore, in light of the global situation, rates of *S*. *agalactiae* resistance in China require ongoing close surveillance to ensure that antibiotic-based prevention and cure for *S*. *agalactiae* infection remain appropriate and safe. We expect that advances in the research of this infection will facilitate the future application of an *S*. *agalactiae* vaccine.

## Conclusion

Our data have revealed the epidemiological characteristics of *S*. *agalactiae* in Beijing. A low *S*. *agalactiae* colonization rate was determined in pregnant women in our study. Serotype III was the most prevalent serotype. All *S*. *agalactiae* isolates were susceptible to penicillin and ceftriaxone and highly resistant to erythromycin and clindamycin. More than 90% of the serotype III/ST19 isolates were resistant to levofloxacin. The multidrug resistance rates of the *S*. *agalactiae* isolates were extremely high, with the highest rate observed for ST19/serotype III.

A limitation of this study is the low number of *S*. *agalactiae* isolates obtained, which may have caused slight deviations in the results. However, considering the general agreement of our results with those of other studies conducted during the same period on serotype distribution and antibiotic susceptibility patterns, our study is valuable as a reference for analyses of the epidemiological characteristics of *S*. *agalactiae* in Beijing, China.
